# Role of the Main Non HLA-Specific Activating NK Receptors in Pancreatic, Colorectal and Gastric Tumors Surveillance

**DOI:** 10.3390/cancers12123705

**Published:** 2020-12-10

**Authors:** Elisa Ferretti, Simona Carlomagno, Silvia Pesce, Letizia Muccio, Valentina Obino, Marco Greppi, Agnese Solari, Chiara Setti, Emanuela Marcenaro, Mariella Della Chiesa, Simona Sivori

**Affiliations:** 1 Centro di Eccellenza per la Ricerca Biomedica, University of Genoa, 16132 Genoa, Italy; elisa06@alice.it; 2 Dipartimento di Medicina Sperimentale (DIMES), University of Genoa, 16132 Genoa, Italy; simona.carlomagno@unige.it (S.C.); silvia.pesce@unige.it (S.P.); letiziamuccio@hotmail.it (L.M.); valentinaobino@gmail.com (V.O.); marcogreppi92@gmail.com (M.G.); agnese.solari@edu.unige.it (A.S.); settichiara.90@gmail.com (C.S.); 3 Dipartimento di Medicina Sperimentale (DIMES) and Centro di Eccellenza per la Ricerca Biomedica, University of Genoa, 16132 Genoa, Italy; mariella.dellachiesa@unige.it

**Keywords:** human NK cells, NKG2D, NCR, DNAM-1, tumor escape, PDAC, CRC, GC

## Abstract

**Simple Summary:**

Natural killer (NK) cells are innate lymphocytes that play a key role in the anti-tumor response by their ability to recognize and kill transformed cells. Their relevance in anti-tumor immune surveillance is demonstrated by the various strategies that tumor cells develop to switch off NK-mediated responses, often aimed at diminishing activating NK receptor function. In this review, we describe the expression and role of the main activating NK receptors displayed by circulating NK cells and/or tumor-infiltrating NK cells in patients with solid tumors of the gastrointestinal tract that affect the pancreas, stomach, and colon-rectus. These malignancies are characterized by high incidence and low survival rate at advanced stages, thus novel therapeutic approaches are urgently needed. We suggest that the most promising immunotherapies are those aimed at restoring activating NK cell receptors dysfunction and overcoming immune suppression exerted by the tumor microenvironment.

**Abstract:**

Human NK cells can control tumor growth and metastatic spread thanks to their powerful cytolytic activity which relies on the expression of an array of activating receptors. Natural cytotoxicity receptors (NCRs) NKG2D and DNAM-1 are those non-HLA-specific activating NK receptors that are mainly involved in sensing tumor transformation by the recognition of different ligands, often stress-induced molecules, on the surface of cancer cells. Tumors display several mechanisms aimed at dampening/evading NK-mediated responses, a relevant fraction of which is based on the downregulation of the expression of activating receptors and/or their ligands. In this review, we summarize the role of the main non-HLA-specific activating NK receptors, NCRs, NKG2D and DNAM-1, in controlling tumor growth and metastatic spread in solid malignancies affecting the gastrointestinal tract with high incidence in the world population, i.e., pancreatic ductal adenocarcinoma (PDAC), colorectal cancer (CRC), and gastric cancer (GC), also describing the phenotypic and functional alterations induced on NK cells by their tumor microenvironment.

## 1. Introduction

Natural killer (NK) cells are innate lymphocytes that can mediate powerful responses against cancerous or virally infected cells. Indeed, they are both “serial killers”, capable of killing multiple targets without requiring any prior antigen exposure, and efficient producers of cytokines important for regulating the activity of additional innate immune cells as well as for influencing the responses of the adaptive immune cells [1,2,3].

To acquire powerful and effective functional properties against tumor and infected cells, but at the same time to ensure self-tolerance, NK cells undergo a process called “licensing” or “education” [4]. This event is necessary because, during the NK cell differentiation/maturation process, each NK cell randomly acquires HLA class I-specific inhibitory receptors (such as inhibitory KIRs, CD94/NKG2A, LILRB-1), and the HLA molecules recognized by these receptors are inherited independently of the receptor genes. As a consequence of the education process, only NK cells expressing inhibitory receptors specific for self HLA class I molecules acquire full functional potential, while the “unlicensed” NK cells are maintained in a state of hypo-responsiveness in order to avoid self-reactive responses (“disarming” hypothesis) [5]. For this reason, the mature and functional NK cells usually express at least one inhibitory receptor, specific for self HLA class I molecules. In contrast, in pathological conditions, virally infected or tumor-transformed cells usually lack/down-regulate the expression of HLA class I molecules (“missing-self hypothesis”) [6], to avoid attack mediated by CD8+ T cells, and acquire/increase the expression of the ligands for the non-HLA specific activating NK receptors (such as NCRs and NKG2D), thus allowing the NK cell-mediated killing of the diseased cells. However, tumor cells can maintain the expression of HLA-I molecules as an immune escape mechanism. Thus, KIR/NKG2A may represent molecular checkpoints capable of limiting NK cell activation against HLA-I^+^ tumor cells. In order to restore the full NK cell activity and functions, recent approaches based on monoclonal antibody (mAb)-mediated blocking of these molecular checkpoints have been developed [7,8]. Preliminary results in ongoing trials have demonstrated a clinical benefit of these approaches, with encouraging response rates [9].

Notably, NK cells can also induce the antibody-dependent cell-mediated cytotoxicity (ADCC) [10] by the engagement of CD16, the low affinity receptor for the Fc fragment of IgG. This capability of NK cells is exploited in several therapeutic approaches in order to redirect a potent NK-cell response against tumor cells. Notably, genetic polymorphisms are known to exist in human CD16 [11,12] that can influence the clinical anti-tumor effects of mAbs. Moreover, CD16 surface expression on NK cells can be downregulated by the action of IL-18 and TGF-β ytokines that often characterize the tumor microenvironment (TME) [13,14]. Importantly, it has been shown that even the metalloproteinase ADAM17 can turn down the CD16 surface expression [15]. However, with the aim to preserve CD16 expression, pharmacological inhibitors and antibodies blocking ADAM17 activity may be used. In addition, interesting innovative approaches to enhance ADCC activity act on optimizing IgG Fc affinity to CD16, primarily when in the presence of lower affinity CD16 polymorphism [10].

Most of the current knowledge on human NK cells comes from studies on peripheral blood (PB)-derived cells (“conventional” NK cells), but, recently, interest in the characterization of NK cells within other tissues has increased, primarily in pathological conditions [16]. NK cells present in the tissues include both “blood-tissue recirculating” and “tissue resident” NK cells that are characterized by distinct phenotypic profiles. Moreover, the TME can influence/induce the de novo expression of immune checkpoints on tumor-associated NK cells as well as reduce the activating NK receptors function or limit the number of tumor-infiltrating NK cells, thus impairing the antitumor response and favoring the tumor progression [17].

Thus, the development of immunotherapeutic approaches capable of harnessing/reconstituting the high potential of NK cells and favoring their expansion in the patients may represent promising antitumor therapy.

## 2. Non-HLA-Specific Activating NK Receptors

Human NK cells express a large array of activating receptors and co-receptors that provide the “on” signal upon interaction with specific ligands on cancer cells. The main activating receptors are able to mediate a powerful activating signal and are represented by NCRs, namely NKG2D and DNAM-1. In contrast, activating co-receptors (including 2B4, NTBA, and NKp80) play a complementary or synergistic role with the main activating receptors in inducing NK cell activation [3].

### 2.1. Natural Cytotoxicity Receptors (NCRs)

The main non-HLA-specific activating NK receptors are represented by the natural cytotoxicity receptors (NCRs) [18], in particular NKp46/CD335 [19,20], NKp44/CD336 [21], and NKp30/CD337 [22], which are encoded by the genes, *NCR1, NCR2*, and *NCR3*, respectively.

First, the NCRs were described as receptors expressed only on NK cells, but later the NCRs expression also on Innate Lymphoid Cells (ILCs), adaptive Vδ1^+^ and CD8^+^ T cells, was shown [23]. Regarding human NK cells, NKp46 and NKp30 are present on the surface of virtually all resting cells, are upregulated upon cell activation, and are downregulated in “adaptive” NK cells of HCMV^+^ individuals and in fully mature NK cells de novo expressing the PD-1 immune checkpoint receptor [24,25]. In contrast to these NCRs, at steady state, NKp44 is expressed only on CD56^bright^ NK cells, even if at low density, whereas upon cell activation with cytokines, it is acquired by essentially all NK cells.

NCRs are type I transmembrane molecules belonging to the Ig superfamily. Their transmembrane domain contains a positively-charged amino acid which is crucial to mediate NCR association with the ITAM-bearing proteins essential for the activation of the NK cell function, in particular CD3-ζ and/or FcεRI-γ (for NKp30 and NKp46) and KARAP/DAP12 (for NKp44) [18]. In contrast to NKp46 and NKp30, NKp44, in its cytoplasmic tail, also contains a sequence that conforms to the immunoreceptor tyrosine-based inhibitory motif (ITIM). This ITIM-like motif lacks inhibitory capacity and is unable to recruit the phosphotyrosine phosphatases, SHP-1 or SHP-2, or the 5′-inositol phosphatase, SHIP [26].

**Figure 1:**
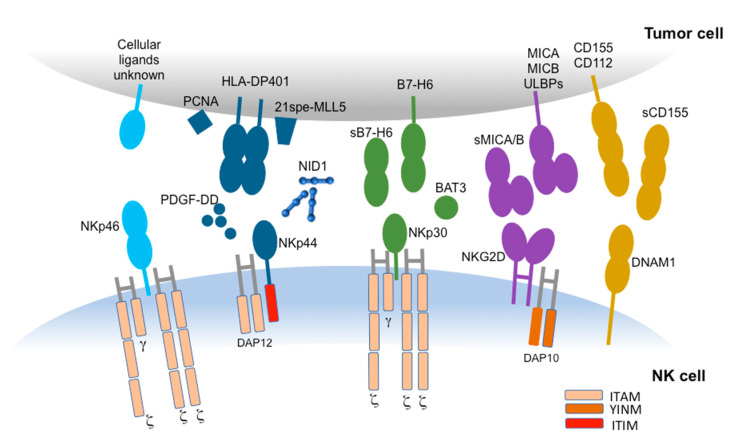
The main non HLA-specific activating NK receptors and their ligands expressed by tumor cells. The main non HLA-specific activating NK receptors described in the text are reported together with their respective ligands, expressed on the surface of cancer cells. The adaptors molecules responsible of signal transduction upon receptor engagement are also depicted. BAT3 is located intracellularly and can be released in a soluble form or expressed on the surface of tumor cells (see text).

Interestingly, the NCRs surface density on NK cells has been shown to correlate with the level of NK-mediated cytotoxicity [27], and even the expression level of NCRs ligands on target cells influences NK cell-mediated response [28]. Unfortunately, so far, only some NCR ligands have been identified. However, some experimental evidences have suggested that each NCR may interact with a wide range of ligands, possibly expressed in different combinations on the surface of the distinct tumor cell types, or released/shed extracellularly, or present in the extracellular matrix (Figure 1). Some of the known NCRs ligands (such as the influenza virus HA, recognized by NKp46 or NKp44) allow for the NK-mediated recognition of virus-infected cells, while others (such as BAT3/BAG6 and B7-H6, ligands for NKp30, or the isoform of mixed-lineage leukemia protein-5, termed 21spe-MLL5, and PCNA, ligands for NKp44) allow for the NK-mediated recognition of tumor cells. In particular, on tumor cells, NCRs may recognize intracellularly localized proteins, such as BAT3/BAG6 and PCNA [29,30,31], that may be expressed at the cell surface of stressed or cancer cells, or cell surface molecules, such as B7-H6 and 21spe-MLL5 [32,33]. NKp46 and NKp44 may also recognize extracellular ligands. In particular, NKp46 can recognize the soluble plasma glycoprotein called complement factor P/properdin [34], whereas NKp44 can interact with Nidogen-1/Entactin [35]. NKp44 has been shown to recognize also a specific HLA-DP molecule (HLA-DP401) [36].

The engagement of NCRs, primarily NKp30, can also induce the release of the chemotactic form of HMGB1, thus amplifying the antitumor response by attracting additional NK cells at the site of tumor-NK cell interaction [37].

Unfortunately, hypoxia and soluble factors produced by tumor/tumor-associated cells (such as IDO-derived L-kynurenine, TGF-β, and PGE2) [38,39,40,41] and soluble forms of NCR-ligands shed from tumor cells surface (such as the soluble form of either BAT3 or B7-H6) [30,42,43] can impair NCR expression and function. Thus, a NCR^low^ phenotype can be observed primarily on tumor-associated NK cells in patients affected by solid and hematologic tumors [44,45].

### 2.2. DNAM-1

DNAX accessory molecule-1 (DNAM-1/CD226) is an activating receptor expressed on NK cells, CD8^+^ T cells, some CD4^+^ T cells, and some myeloid cells [46,47,48]. DNAM-1 is an adhesion and costimulatory molecule that can trigger NK cell cytotoxicity and mediate interferon gamma (IFN-γ) release upon recognition of CD112 (nectin-2) and CD155 (poliovirus receptor) which are expressed on a broad range of cells, including transformed cells and virus-infected cells [49]. The same ligands can be recognized also by TIGIT and CD96 immune checkpoints that, differently from DNAM-1, can mediate the inhibition of NK cell functions. DNAM-1 also has a key role in expansion and maintenance of adaptive NK cells at least in mice [50].

Notably, in healthy donors, most human PB NK cells express DNAM-1, whereas in oncologic patients, DNAM-1^neg^ NK cells, characterized by limited effector functions, have been described [45,51,52]. In this regard a key role is played by soluble forms of PVR and Nectin in TME [53,54].

### 2.3. NKG2D

NKG2D is a type II transmembrane and C-type lectin-like receptor that associates with the transmembrane adaptor protein DAP10 to transduce activating signals. It is expressed on NK cells, but it may be expressed also on cytotoxic T cells.

NKG2D ligands are represented by HLA class I structural homologues, called ULBPs and MICA/B, that are upregulated in infected, stressed, and tumor cells [55,56,57]. The NKG2D ligands are usually expressed in most epithelial-derived tumor cells, such as ovarian cancer, colon cancer, and leukemia, but they have been rarely detected at low levels in healthy adult tissues, such as gastrointestinal epithelial cells [58]. Unfortunately, in oncologic patients, NKG2D function can be impaired by soluble forms of its ligands that can be shed by tumor cells [59].

Radiation therapy (RT) and different types of therapeutics agents can favor the expression of NKG2D ligands on the surface of tumor cells. Sub-lethal doses of genotoxic chemotherapeutics induce upregulation of NKG2D ligands, at both the protein and mRNA levels. The heat shock 90 kDa protein (HSP90) inhibitors activate heat shock transcription factor 1 (HSF1), a powerful enhancer of MICA/MICB transcription. The pharmacological inhibition of glycogen synthase kinase 3 (GSK3) significantly decreases the constitutive phosphorylation of signal transducer and activator of transcription 3 (STAT3), a negative regulator of MICA transcription [60]. The histone deacetylase (HDAC) inhibitors (HDACIs) not only increase the expression of NKG2D ligands on tumor cells, but also reduce their shedding from the surface of tumor cells [61].

## 3. Gastrointestinal Cancers

Gastrointestinal cancers include several malignancies of the gastrointestinal tract and accessory organs such as the stomach, liver, intrahepatic bile duct, and pancreas. Despite the fact that all of them have an epithelial cell origin, there are great differences in the incidence and prognosis among the component sites. In this review, we focus on three digestive malignancies that are among the most common cancers worldwide, i.e., pancreatic ductal adenocarcinoma (PDAC), colorectal cancer (CRC), and gastric cancer (GC). Disease progression in these cancers has been associated with tumor immunosurveillance escape. In particular, as observed in several cancer types [62], NK cell dysfunction has been described in all three cancer types.

### 3.1. PDAC

Pancreatic ductal adenocarcinoma (PDAC), that is the most common histological type of pancreas malignancies, is a devastating disease with a five-year survival rate below 5%. It is characterized by rapid progression and intrinsic and acquired drug resistance [63], and exhibits aggressive growth and early metastatic dissemination. Since no clinically informative early diagnostic symptoms and biomarkers are available, most patients are identified too late for treatment by curative surgical resections, which represent the only chance for cure. Different factors, including anatomical location of the tumor, delayed diagnosis, and chemo-resistance amplified by the stromal barrier localized around the tumor, play a crucial role in contributing to the poor prognosis observed in patients affected by PDAC [64,65].

A hallmark of PDAC is the strong desmoplastic reaction which occurs in the TME resulting in a dense fibrotic/desmoplastic stroma that surrounds the pancreatic cancer cells, composed of cellular and acellular components, including fibroblasts, immune cells, pancreatic stellate cells (PSCs), endothelial cells, extracellular matrix, and soluble proteins such as cytokines and growth factors. Conversely, immune cells mediating anti-tumor effects, like DCs, NK cells, and CD8^+^ T cells, are relatively few and their anti-tumor effects are generally impaired [64,66,67].

The progression of pancreatic cancer is significantly promoted by tumor immune escape, resulting from the dysfunction of multiple immune cells, including NK cells and natural killer T (NKT) cells [68].

One of the mechanisms for tumor to escape the control of the immune system is the capacity to shift NK cells from activation to a hyporesponsive state by downregulating the expression of several activating receptors [69]. It has been shown that progression of pancreatic cancer is closely associated with dysfunctional circulating NK cells in PB [69]. However, the presence of NK cells infiltrating the pancreatic tumor and the analysis of NK cells role in limiting PDAC progression need to be investigated more thoroughly. Although the high number of circulating NK cells in PDAC patients positively correlate with survival, the cytotoxicity of PDAC-associated NK cells was impaired compared to those of healthy donors. It has been recently demonstrated that PDAC tissues present a very low frequency (<0.5%) of NK cell infiltrate [65]. Lim and colleagues suggested that this low frequency could be a unique feature of patients with pancreatic cancer, as tumor-infiltrating lymphocytes (TILs) isolated from patients with other solid tumor malignancies show higher percentage of NK cells. The idea of this study is that circulating NK cells lose the surface expression of CXCR2, rendering them incapable of trafficking toward PDAC. Moreover, circulating NK cells in these patients do not express CXCR4, and its ligand was not produced by PDAC cells, suggesting that no chemokine signals could attract circulating NK cells within the tumor tissue [65].

### 3.2. CRC

Colorectal cancer (CRC) is the third most commonly diagnosed cancer in the world. A mixture of genetic and epigenetic modifications contribute to CRC etiology [70]. The molecular mechanisms described in the CRC carcinogenesis pathways included chromosomal instability, microsatellite instability, aberrant DNA hypermethylation and defects in DNA repair [71,72] that give rise to a high heterogeneity of tumor genetic patterns and clinical outcomes. Surgery intervention represents the ideal treatment to achieve complete removal of the tumor and metastases, however nearly a quarter of CRC are diagnosed at an advanced stage and, despite surgical resection of the tumor, a substantial number of cases develops recurrence or dissemination [73]. In the last few years, the “immunoscore”, that is the analysis of the immune profile in the tumor area, has been proposed and validated as prognostic marker in CRC [74,75]. Indeed, the presence of TILs have been shown to be correlated with the containment of metastases [76] and with a good clinical outcome [77]. NK cells infiltrating CRC microenvironments, together with CD8^+^ T cells, has been associated to a better prognosis of the disease [78,79]. At this regard, an in vitro study suggested that NK cells might produce IFN-γ in an NKG2D-dependent manner and favor T cell differentiation toward a Th1 profile [80]. In contrast to the frequently observed shortage of NK cells in solid tumor, in the PB of CRC patients, the number of circulating NK cells is shown to be higher than in healthy individuals and progressively increase in relation to metastatic progression [81]. However, PB NK cells from CRC patients present an altered receptor repertoire and multiple dysfunctions [69,81,82].

### 3.3. GC

Gastric cancers (GC), with gastric adenocarcinoma as the prevalent histological type, are very common neoplasms with high incidence of cancer death. GC is characterized by high metastatic frequency as it tends to be diagnosed late, with no major symptoms at early stages [83]. The incidence of gastric cancer varies widely depending on genetic and environmental factors and also by geographic region. Among the several environmental risk factors identified for GC, infection by the bacteria *Helicobacter pylori* in the stomach appears to be a major cause of this type of cancer [84]. In addition, GC is also associated with infection with Epstein–Barr virus in a particular GC subtype, identified by the TCGA classification [85].

Similar to PDAC and in general to most solid tumors, the TME plays a key role for GC progression, metastasis, and therapeutic response. Among immune cells populating the TME in gastric cancer, NK cells are likely involved in tumor control and progression. Interestingly, the abundance of NK cells in the TME resulted in a robust prognostic marker associated to the best prognosis compared to other TME features (such as soluble factors, T cells, or endothelial cell contents) in a large cohort of GC cases [86]. In line with this result, various studies have already shown that decreased NK cell infiltration in tumor tissues from GC biopsies correlated with lower survival rates and disease progression [87,88]. As suggested for PDAC, the reduced NK cell infiltration could be a consequence of either a decreased migratory capacity of NK cells or enhanced apoptosis in response to local factors of NK cells that infiltrate tumors. On the other hand, NK cell numbers are reduced also in PB from GC patients and can be restored after surgical resection [89,90].

## 4. NCRs Role in PDAC, CRC and GC

### 4.1. PDAC

The expression of NK activating receptors, like NKp30 and NKp46, in the PB NK cells of PDAC patients is significantly reduced, consistent with studies in other malignancies, such as cervical cancer, breast cancer, peritoneal carcinomatosis, ovarian cancer, and melanoma [42,45]. This lower number of cells expressing NKp30 and NKp46 may be one of the mechanisms responsible for the poor function of NK cells in these patients. Peng and colleagues demonstrated that the expression of NKp30 and NKp46 correlated with pathological stage and histological grade in patients with PDAC, as well as GC and CRC (see below), which indicates that NK cell dysfunction may participate in malignant progression in these tumor types. Different pro-tumorigenic factor produced by cancer cells are capable of inducing NK cell dysfunction to escape the attacks from immune system. For example, in PDAC patients the production of indoleamine-2,3-dioxygenase (IDO) and MMP-9 from cancer cells can significantly limit the cytotoxicity of NK cells, inducing anergic/hypofunctional NK cells in vitro through decreasing the expression of NKp30, NKG2D, and perforin, as well as inhibiting the secretion of IFN-γ and tumor necrosis factor (TNF-α) [63,69].

### 4.2. CRC

Reduced functionality of NK cells mediated by NCRs impairment has also been described in CRC patients.

In healthy gut mucosal tissue, NKp46^+^ cells are not strictly associated with lymphoid aggregates but are rather mainly detectable within lamina propria of the ileum villi and colonic crypts. The intraepithelial position of NK cells, certainly included in NKp46^+^ cells, gives them the chance to interact with intestinal microorganisms and antigen presenting cells and, consequently, the possibility to respond directly to pathogenic microorganisms or to collaborate to shape a subsequent adaptive T cell response [91,92]. In this regard, human NK cells have also been demonstrated to be involved in the recognition of *Fusobacterium nucleatum (F. nucleatum)*, an oral commensal Gram-negative anaerobic bacteria, whose presence in the gut has been associated with the development of CRC. Indeed, several studies demonstrate that *F. nucleatum* participates in the maintenance or induction of inflammatory microenvironments, promoting tumor cell growth and metastasis [93,94,95]. *F. nucleatum* can directly interact with human NK cells through the activating receptor NKp46 [96] and the inhibitory receptor TIGIT [97]. However, this dual interplay doesn’t have beneficial effects. Rather, it seems to promote the progression of CRC and the impairment of NK cell response since the engagement of NKp46 by *F. nucleatum* induces TNF-α production, which could worsen inflammation in the mucosa, whereas TIGIT mediated signaling inhibits NK cell cytotoxicity. Moreover, TIGIT expression on tumor-infiltrating NK cells is associated with tumor progression in tumor-bearing mice and patients with colon cancer and it is linked to the functional exhaustion of NK cells [98].

The scarce amounts of NK cells in CRC tissue from the early stage, compared to NK cells amounts in non-malignant colon tissue, had limited a deeper analysis of the rare NK cell infiltrate in terms of receptor repertoire and functional capabilities [78,99,100]. In 2012, Rocca and coworkers displayed for the first time that NK cells infiltrating human CRC tissue displayed a profound alteration of their phenotype with a drastic reduction of different NK cell receptor expression, including NKp46 and NKp30, with respect to NK cells infiltrating normal mucosa and NK circulating in PB derived from the same donors [100]. NKp44 expression instead was increased both in tumor and normal tissue. However, NKp44 and NKp46 could be also expressed by ILCs [101,102,103,104,105], implicated in inflammatory responses, and described in substantial numbers in CRC where they’re supposed to be implicated with the initiation and the evolution of the disease [106,107,108]. Different mechanisms such as a chronic exposure to receptor ligands or cytokine milieu could explain the down-regulation of these activating receptors that might be associated with a loss of NK cell functions in situ.

On the other hand, it has been demonstrated that CRC-derived cancer initiating cells (CICs) express NKp30 and NKp44 ligands at higher levels than non-CICs CRC cells and these NCRs ligands have an important dominant role in driving autologous NK cell mediated killing of CRC-derived CICs. Non-CIC counterpart showed less susceptibility to PB derived NK cells lysis due not only to a reduction of NCR-ligands but also to a higher expression of HLA class I molecules that co-localize in the membrane at the same region of NCRs ligands. The high expression of inhibitory ligands and low expression of activating ligands may contribute to the relative resistance of the more differentiated CRC cells to NK cell lysis, and vice versa, the high expression of activating ligands and low expression of inhibitory ligands may suggest NK cells as a possible player eliminating CRC-CICs, limiting the disease burden [109].

Interestingly, the infiltration of the CRC microenvironments by NK cells, together with CD8^+^ T cells, has been associated to a better prognosis of the disease [78,79].

Different studies analyzed the amount and the phenotype of NK cells derived from PB of CRC patients [110,111,112,113] and agreed on a compromised NCRs expression. Indeed, in CRC patients, the percentages of NK cells expressing NKp46 and NKp30 are significantly lower than in healthy controls, as well as the percentage of perforin positive NK cells. [69,100]. In addition, the down-regulation of NKp46 receptor expression seems to be correlated with lower relapse-free survival (RFS) of CRC patients with a follow-up more than five years [81]. According to NKp46 and NKp30 down-modulation, circulating NK cells derived from CRC patients present a lower lytic response against CRC cell lines and reduced cytotoxic capacity and IFN-γ secretion when co-cultured with NK susceptible target cells, such as K562 [81].

### 4.3. GC

In keeping with what has been observed in PDAC and CRC, a downregulation in the expression of the main NCRs, i.e., NKp30 and NKp46, together with NKG2D and DNAM1 (see below), has been observed in circulating NK cells from GC patients at different stages. A possible underlying mechanism is based on TGF-β release by tumor cells, because of its high serum level characterizing GC patients that is negatively correlated with the frequency of NK cells expressing NKp46, NKp30 but also NKG2D and DNAM1 [114]. As expected, this impaired expression of NKp30 and NKp46 is associated to tumor progression. In particular, NKp30 downregulation on PB-NK cells also correlates with depth of invasion [69]. However, the actual role of NKp30 in controlling GC is unclear considering that the expression of its main ligand B7-H6 on GC biopsies showed no prognostic significance [115]. On the other hand, few data are available on NCRs expression by tumor infiltrating NK cells and further investigations are needed also to disclose different mechanisms underlying NCRs downregulation [88]. In this context, the downregulation of the signaling protein CD3ζ was observed in PB-NK cells from GC patients and affected CD16-mediated responses to Herceptin (anti-HER2 mAbs) [116], but also possibly dampened surface expression and signaling through NKp46 and NKp30.

## 5. NKG2D Role in PDAC, CRC and GC

### 5.1. PDAC

It was recently demonstrated that NK cells exhibit impaired cell-mediated killing of autologous PDAC cells due to the insufficient ligation to NKG2D, another NK activating receptor [65].

NKG2D expression can be affected by a variety of factors, including changes in TME soluble factors. Malignant cells adopt different strategy to reduce the levels of NKG2D ligands (MICA/B and ULBP1–6) and escape NKG2D-mediated immune surveillance [117]. An evasion mechanism for immune surveillance of pancreatic tumor cells could be the shedding of MICA/B into the TME and serum.

Published data have demonstrated that the membrane bound form of MICA was prevalently expressed in the low-grade cancers, whereas higher levels of the soluble form (sMICA) were detected in the sera of patients with advanced PDAC, suggesting a correlation with the progression of the disease and the distant metastasis [118]. A significant inverse correlation between serum levels of sMICA and levels of NK cell surface NKG2D expression was observed in all patients of this study. Moreover, sMICA released in the serum of PDAC patients may interact with other soluble mediators, such as immune-related or pro-tumorigenic cytokines produced by tumor cells, which can affect the development and progression of PDAC. sMICA inducing the impairment of NK cell antitumor immunosurveillance may be involved in angiogenesis and/or tumor growth either directly or indirectly through close relationships with corresponding cytokines that are engaged in these functions as well as down-regulation of antitumor immunity in PDAC [63,69,118,119,120,121].

The metalloproteinases ADAM 10 and ADAM 17 are involved in the mechanism that causes the shedding of MICA/B in tumor cells, followed by internalization and degradation upon binding to NKG2D [119,121,122].

A positive restoration of NK cell activation was demonstrated in the chemotherapeutic treatment of PDAC mouse models. In these experiments the gemcitabine mediated anti-tumor effects was due also to an increase of NK cell infiltration in the tumor tissue and a downregulation of myeloid suppressor cells [123,124]. In another study, low doses of gemcitabine have been shown to induce a MICA/B upregulation on cells membrane and enhance innate immune function rather than cytotoxicity in pancreatic cancer. In addition, these data suggest that the inhibition of cleavage and release of MIC molecules from the tumor surface could potentially improve NKG2D-dependent cytotoxicity [124].

Notably, the preferential expression of MICA/B on cancer stem cells (CSCs) from PDAC cell lines and dissociated primary cancer samples was described to favor the NKG2D-dependent killing of CSCs by NK cells [125].

### 5.2. CRC

Similar to what was demonstrated for NCRs, in CRC patients, NK cells expressing lower proportion of NKG2D have been described in tumor infiltrating lymphocytes and in PB [69,81,100,126].

The decrease of circulating NK cells expressing NKG2D and perforin is correlated with the histological grade in CRC and it is more pronounced in patients with lymph node metastasis [100]. Impairment of NKG2D expression could be dependent on two tumor escape mechanisms blinding activity both on NK cells and CD8^+^ T cells: the higher amounts of TGF-β [39,81] and the higher concentration of sMICA [59,127] into the plasma of CRC patients in comparison to plasma of healthy individuals.

However, higher MICA expression levels on CRC cells have been correlated with improved disease-specific survival and good prognosis [128,129]. In this regard, a recent study, collecting patients from early to metastatic stages, showed that, in CRC lesions, the level of miR-20 is increased. Increment of this regulatory RNA sequence seems not to have effects on tumor cell proliferation but rather on expression of MICA at cell surface. Indeed, miR-20 overexpression is negatively correlated with MICA expression on CRC cells and causes the impairment of NKG2D-mediated killing by circulating NK cells, suggesting an additional immune surveillance escape mechanism [130].

Finally, a recent genetic study associated the MICA*012:01 allele with KRAS codon 12 mutation and it is suggested as a negative prognostic value since CRC patients with MICA*012:01 seem to have a tendency to experience relapse or metastasis in the first 20 months after surgery [131].

### 5.3. GC

NKG2D plays a pivotal role in NK-mediated recognition and killing of GC as well [132]. This could be expected considering NKG2D ligands expression on GC [133] and it is suggested also by a better overall survival (OS) in patients with higher levels of NKG2D expression compared to patients with lower/no expression of NKG2D in TILs [133,134]. On the other hand, tumor-infiltrating CD56^+^ T cells expressing NKG2D could also contribute to this favorable effect [133]. Notably, NK cells can also kill gastric CD133^+^ cancer stem cells via NKG2D, thus suggesting their possible role in controlling GC relapses/progression [135]. However, a decreased NKG2D expression has been documented on both PB and tumor infiltrating NK cells in advanced GC patients [114,136] and has been correlated to LN metastases and blood vessel invasion [69]. As already described in PDAC and CRC, both sMICA/B and TGF-β appear responsible for NKG2D weakening on patients NK cells [114,137]. Indeed, MMP-9 expression was inversely correlated to NKG2D ligands expression in GC biopsies, possibly leading to lower susceptibility to NK cell mediated control. Interestingly, MMP inhibition restored NKG2D ligands (NKG2D-L) expression in several GC cell lines improving NK cell-mediated killing in vitro [138]. In line with the relevance of NKG2D/NKG2D-L interaction in tumor control there is the recent observation that gastric circulating tumor cells (CTC) undergoing epithelial to mesenchymal transition (EMT) downregulate ULBP1 [139], thus possibly favoring metastatic growth by evading NK-mediated killing.

## 6. DNAM-1 Role in PDAC, CRC and GC

### 6.1. PDAC

In PDAC patients, the surface downregulation of DNAM-1/CD226 has been reported. This downregulation could indicate NK cell dysfunction and may favor PDAC progression [68]. Moreover, the decrease of CD226^+^ NK cells percentage correlated with the tumor histological grade and lymph node metastatic spreading [68].

The downregulation was observed also for CD96, and not for the receptor TIGIT, which share with CD226 and CD96 the same ligand, CD155. Peng and co-authors evaluated also the expression of CD155 in pancreatic cancer tissue by immunohistochemistry. The binding of CD155 with DNAM-1 and CD96 promotes adhesion of the NK cell to the target cell and also the synthesis of cytotoxic granules that induce the target cell lysis [68]. The level of CD155 was significantly higher in tumor cells than in adjacent tissues, pointing towards the potential of therapies that increase CD226 expression on NK cells.

### 6.2. CRC

Significant lower proportion of NK cells expressing DNAM-1 has also been described in CRC patients, both on PB NK cells and tissue infiltrating NK cells [69,81,100,126,140]. Down-modulation of DNAM-1 on PB NK cells could be related to the higher expression of soluble CD155 in serum of CRC patients and could finally led, together with sNKG2D-Ls, to the inhibition of NK cytotoxicity. At this regard, high nectin-2 serum levels at early stage of CRC disease have been suggested as bad prognostic factor for patient survival [141]. Also, CRC tissue expresses DNAM-1 ligands at a higher level than peritumoral normal mucosa and, in particular, CD155 is more up-regulated than CD112. Expression of DNAM-1 on NK cells infiltrating CRC is deeply weaker than on circulating NK cells derived from the same donor [100].

In vitro studies of NK cells co-culture with CRC lines, expressing DNAM-1 ligands, showed that the physical contact is needed for DNAM-1 down-modulation [100] and suggest that the down-modulation of DNAM-1 can depend on the expression of receptor ligands in vivo.

### 6.3. GC

Downregulation in DNAM-1 expression was observed on circulating NK cells from GC patients [69,114] similar to what observed on PDAC and CRC patients. However, negligible differences in DNAM-1 expression were observed between NK cells infiltrating tumor and non-tumor tissues in a single study [88]. Further studies are needed in view of the putative expression of DNAM-1 ligands on GC based on genomic data reported by TCGA [132].

## 7. Conclusions

The central role of NK cells in controlling tumor growth and in mediating a robust anti-metastatic function has been demonstrated in several preclinical studies regarding solid tumors, including gastrointestinal cancers. The high density of tumor-infiltrating NK cells has been associated with a good prognosis in CRC and GC.

**Figure 2:**
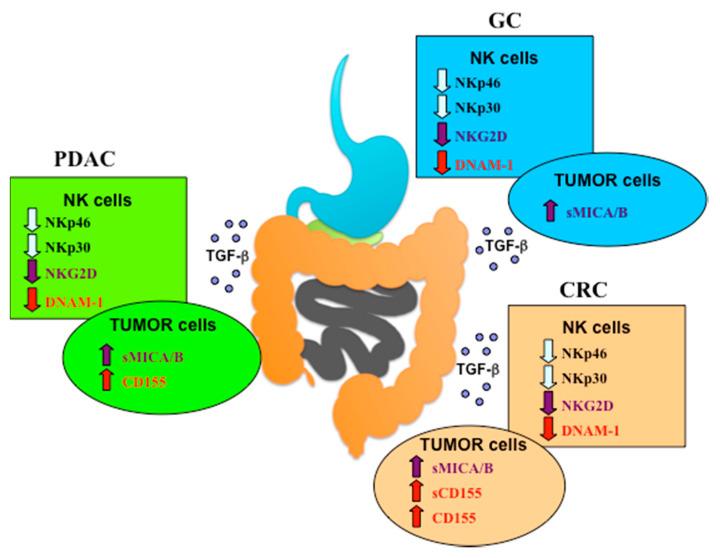
Activating NK receptors are downmodulated in NK cells from PDAC, CRC and GC patients. The activating NK receptors indicated in the boxes are downregulated on NK cells in most patients affected by the different gastrointestinal tumors. Matched colored arrows indicate receptors/ligands pairs. The mechanisms underlying this downmodulation, shared by NK cells from PDAC, CRC and GC, are related in most cases to TGF-β production or to ligands shedding from tumor cells, in particular soluble MICA/B (sMICA/B) from all three cancer types and soluble CD155 (sCD155) from CRC.

On the contrary, the immune evasion mechanisms developed by the TME of PDAC, CRC and GC may result in the downregulated expression of the activating NK receptors or in the shedding of their ligands, events that are associated with poor prognosis and spreading of metastasis. TGF-β, a key immunosuppressive cytokine produced by tumor cells, stromal cells, Treg, and myeloid-derived suppressor cells, plays a relevant role in the EMT process and cancer progression as well as in the suppression of activating NK receptor expression and NK-mediated antitumor response in advanced cancer patients (Figure 2).

Much progress has been made regarding the role of NK cell checkpoints in anti-tumor immunity. Checkpoints targeting has displayed the capability of harnessing NK cell activity against tumors. However, a lot remains to be understood. In particular, further investigations are necessary to know in more detail the features of tumor-infiltrating NK cells. Do they represent cells that are exhausted or simply inhibited by local TME conditions? Furthermore, is it sufficient to block the checkpoints or is it also necessary to increase the expression of activating NK receptors?

Immunotherapeutic strategies aimed at restoring and harnessing the NK cell function against gastrointestinal cancers, including the adoptive transfer of unmodified and genetically modified NK cells, are currently employed in preclinical and clinical studies. The increasing knowledge on the ex vivo NK cell expansion/activation methods and the currently available approaches to genetically modify NK cells, are paving the way to promising therapeutic NK-based approaches that could also become powerful weapons in the fight against these awful solid tumors. Indeed, producing large numbers of cytotoxic NK cells and improving the function of NK cells by their ex vivo activation or chimeric antigen receptor (CAR) modification are relevant tools for a successful adoptive immunotherapy. The genetic modification of NK cells with CAR targeting different tumor-associated antigens, including EpCAM [142,143], MUC-1 (in CRC, GC, pancreatic cancer; NCT02839954), NKG2D-L (in metastatic solid tumors; NCT03415100), and ROBO1 (in pancreatic cancer; NCT03941457), have been developed with exciting results. Bi- and Tri- specific killer cell engagers (BiKEs and TriKEs) can represent promising approaches for cancer immunotherapy, complementing existing immune-oncology treatments [144].

Finally, in specific gastrointestinal cancers, such as PDAC, the use of approaches capable of favoring the NK cells crossing through the stromal barrier localized around the tumor should also be considered in order to further increase the efficacy of an NK cell-based adoptive therapy.
